# Supplementary material for: Development of a Novel Recombinant Adeno-Associated Virus Production System Using Human Bocavirus 1 Helper Genes
Source: Mol Ther Methods Clin Dev. 2018 Oct 4;11:40–51. doi: 10.1016/j.omtm.2018.09.005 (PMC6205362; doi:10.1016/j.omtm.2018.09.005)
Supplement: Document S1. Figure S1 [file mmc1.pdf]

OMTM, Volume *11*

Supplemental Information

Development of a Novel Recombinant Adeno-Associated Virus Production System Using Human Bocavirus 1 Helper Genes

Zekun Wang, Fang Cheng, John F. Engelhardt, Ziying Yan, and Jianming Qiu

Supplemental Figure Legends

Fig. S1. Characterization of the biological properties of rAAV2/2 produced with the help of pBocaHelper.

(A) Electronic microscopy. Negative staining of recombinant AAV2 produced with the help of pAdHelper or pBocaHelper. **(B) The transduction efficiency of rAAV2 produced with pAdHelper or pBocaHelper.** HEK293 and HeLa cells were transduced at an MOI of 5,000 gc/cell. The expression of mCherry was viewed under a fluorescence microscope. **(C) Quantitative analysis the transduction efficiency by luciferase assay.** The transduced cells were lysed and the luciferase activities were measured. n.s., statistically no significant.

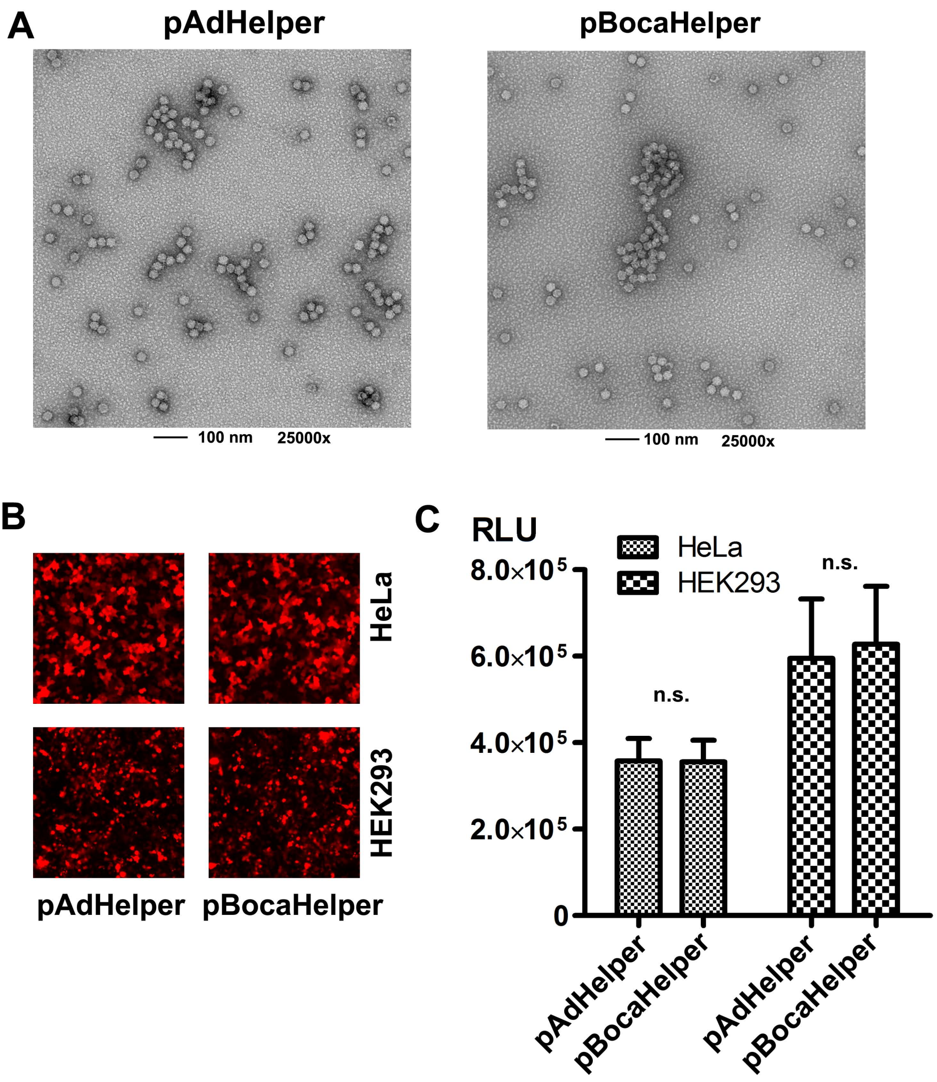
A
pAdHelper
pBocaHelper
100 nm
25000x
100 nm
25000x
B
HeLa
HEK293
pAdHelper
pBocaHelper
C
RLU
HeLa
HEK293
8.0×10^5
6.0×10^5
4.0×10^5
2.0×10^5
0
n.s.
n.s.
pAdHelper
pBocaHelper
pAdHelper
pBocaHelper

Figure S1
