# Supplementary material for: Development of a Novel Recombinant Adeno-Associated Virus Production System Using Human Bocavirus 1 Helper Genes
Source: Mol Ther Methods Clin Dev. 2018 Oct 4;11:40–51. doi: 10.1016/j.omtm.2018.09.005 (PMC6205362; doi:10.1016/j.omtm.2018.09.005)
Supplement: Document S2. Article plus Supplemental Information [file mmc2.pdf]

Molecular Therapy
Methods & Clinical Development
Original Article

Development of a Novel Recombinant Adeno-Associated Virus Production System Using Human Bocavirus 1 Helper Genes

Zekun Wang,[1] Fang Cheng,[1] John F. Engelhardt,[2,3] Ziying Yan,[2,3] and Jianming Qiu[1]

[1]Department of Microbiology, Molecular Genetics and Immunology, University of Kansas Medical Center, Kansas City, KS 66160, USA; [2]Department of Anatomy and Cell Biology, University of Iowa, Iowa City, IA 52242, USA; [3]Center for Gene Therapy, University of Iowa, Iowa City, IA 52242, USA

Human bocavirus 1 (HBoV1), an autonomous parvovirus, is a helper virus supporting replication of wild-type adeno-associated virus 2 (AAV2). In this study, we compared the helper functions from HBoV1 with those from adenovirus (Ad) for the production of recombinant AAV (rAAV) vector in HEK293 cells. We demonstrated that triple plasmids transfection of (1) a cloned HBoV1 helper minigenome (pBocaHelper) that expresses HBoV1 genes *NP1*, *NS2*, and *BocaSR*, (2) pAAV transfer plasmid, and (3) pAAVRepCap supports rAAV production in HEK293 cells. Despite a production yield of 1–2 log lower than that using pAdHelper (expressing Ad genes *E2A*, *E4*, and *VA*), rAAV vector produced using pBocaHelper transduced cells as efficiently as that produced using pAdHelper. The low vector production is largely due to the inefficient expression of the AAV Rep52 and capsid proteins, as well as reduced rAAV genome replication. When the AAV capsid proteins and Rep52 were ectopically expressed under strong promoters, the enhanced protein expression significantly improved the rAAV production using pBocaHelper, approaching a level of 50%–70% of that produced using pAdHelper. Through further dissection of the helper functions from pAdHelper in a five-plasmid transfection system, we found that the addition of the Ad *E2A* gene to the above HBoV1 helper system significantly increased rAAV DNA replication, which increased the rAAV vector production to a level of 3–7 times higher than that using pAdHelper. We finally combined HBoV1 *NP1* and *NS2* genes with Ad helper genes to create a novel dual helper plasmid (pABHelper) for rAAV vector production in the conventional three-plasmid transfection system. The pABHelper facilitated rAAV production at a yield ∼2 times higher than that using the pAdHelper.

INTRODUCTION

Adeno-associated viruses (AAVs) are members of the genus *Dependoparvovirus* of the family Parvoviridae.[1] The recombinant AAV (rAAV) vector has emerged as one of the preferred delivery agents for clinical gene therapy. The only *cis* element in rAAV-mediated gene delivery is the inverted terminal repeats (ITRs) at the two ends of the rAAV genome.[2] rAAV genome does not integrate into chromosome; instead, it forms episomes that are responsible for the long-term expression of transgene.[3] Importantly, rAAV can transduce both dividing and nondividing cells efficiently.[4] At least 13 AAV serotypes originating from human and nonhuman primates have been characterized.[5] In addition, many new AAV capsid-coding sequences have been discovered in recent years using the high-throughput sequencing technology.[6] Moreover, direct capsid evolution and rational capsid design have greatly increased the diversity of AAV capsids with improved specificity, efficiency, and escape of neutralizing antibodies.[7,8] Despite the cross-package of rAAV2 genome by AAV serotypes or variants, it can also be packaged cross-genus efficiently, for example, with the capsid of human bocavirus 1 (HBoV1),[9] which extends the toolbox of AAV capsid variants further.

Efficacy, persistence, and safety of the rAAV vector have been observed in preclinical animal model studies and clinics of human gene therapy. Clinical trials using the rAAV vector have yielded positive outcomes.[10–17] In 2017, Luxturna, a rAAV2 vector encoding *RPE65* gene, became the first gene therapy drug for treatment of inherited vision loss to receive marketing approval in the US.[18] The success of rAAV-based gene therapy and increasing number of preclinical and clinical trials demand more rAAV vectors. The clinical doses for rAAV vectors range from 10^{11} to 10^{14} genomic particles per patient dependent on therapeutic areas.[13,19] However, the manufacturing processes for rAAV vector is time consuming, labor extensive, and expensive. Current production capacity cannot provide sufficient rAAV vectors for a large number of patients in late-phase clinical trials and even limit the commercialization of approved therapeutics due to high cost.

To scale up the rAAV production, several methods had been developed, including the stable cell line, baculovirus production system, herpes simplex virus production system, and vaccinia virus production system.[20–23] However, establishment of stable cell lines,

Received 23 July 2018; accepted 27 September 2018;
https://doi.org/10.1016/j.omtm.2018.09.005.

Correspondence: Jianming Qiu, Department of Microbiology, Molecular Genetics and Immunology, University of Kansas Medical Center, Mail Stop 3029, 3901 Rainbow Blvd., Kansas City, KS 66160, USA.
E-mail: jqiu@kumc.edu

 © 2018 The Author(s).
This is an open access article under the CC BY license (http://creativecommons.org/licenses/by/4.0/).

generation of helper viruses, and the limited titer of the helper viruses are time consuming and not flexible. Transient transfection of plasmids into HEK293 cells for the production of rAAV is still the most commonly used method in research labs and in preclinical and the early stage of clinical trials.[22]. In this method, the transfection strategy is free of helper virus contamination and can be scaled up in a large quantity in bioreactors.[24]

AAV is a defective virus; its replication not only relies on host factors but also needs the necessary functions provided by helper virus.[25] Thus far, several helper viruses for AAV have been characterized, including adenovirus (Ad), herpes simplex virus, and vaccinia virus in mammalian cells, and baculovirus in insect cells.[26] The replication of rAAV genome and assemble of rAAV virions in the current helper-free rAAV production system in HEK293 utilize AAV Rep and capsid (Cap) proteins, expressed from an AAV helper plasmid (pAAVRepCap) in *trans* and four Ad helper genes: the *E2A*, *E4*, and *VAI*, which are expressed from one plasmid, e.g., pHelper (pAdHelper), and the *E1* gene, which is integrated in HEK293 cells.[27,28] rAAV produced from this system always yields rAAV 1-log less compared with the wild-type (WT) AAV production from an AAV2 infectious clone, where all Rep and capsid proteins are expressed in *cis*.[29] Therefore, there are still rooms to increase rAAV production from the HEK293 cell transfection system.

For the lytic life cycle of WT AAV infection and the production of rAAV vectors, there are no obvious similarities in terms of the helping functions and/or mechanisms provided by different helper viruses so far characterized. Recently, we identified that an autonomous parvovirus HBoV1 is a novel helper for WT AAV2 replication in polarized human airway epithelial cultures.[30] Two small nonstructural proteins, NS2 and NP1 and the viral long noncoding RNA BocaSR were identified as essential to facilitate AAV2 replication not only in HEK293 cells that are Ad *E1* gene transformed but also in non-*E1* gene-transformed HeLa cells.[30] We hypothesized that rational combination of helper genes from different helper viruses may develop a super helper for rAAV production.

In this study, by rational addition of HBoV1 *NP1* and *NS2* genes into Ad helper gene sets, we developed a novel HEK293 helper system that produces ∼2-fold greater yield than the traditional three plasmid Ad-based helper system.

RESULTS

HBoV1 Helper Facilitates rAAV Vector Production

We have previously found that HBoV1 helped WT AAV2 replication during co-infection in polarized human airway epithelia and that *NP1*, *NS2*, and *BocaSR* genes were identified as the HBoV1 helper genes for full replication of AAV2 in HEK293 cells.[30] We sought to determine whether HBoV1 could similarly complement rAAV production, and if so, to determine the minimal required components. To this end, an HBoV1 minigene helper was constructed. We combined the three HBoV1 genes into one plasmid pBocaHelper (Figure 1A), from which the open reading frames (ORFs) of NP1 and NS2 linked by a porcine teschovirus-1 self-cleaving peptide (P2A) were expressed by the cytomegalovirus (CMV) immediate early (IE) promoter and the BocaSR was expressed under its own internal pol-III promoter.

To test the helper function of pBocaHelper in supporting rAAV production by transfection, HEK293 cells were co-transfected with pBocaHelper, a rAAV2 (proviral) transgene plasmid that hosts mCherry and luciferase expression cassettes flanked by two AAV2 ITRs, and an AAV helper plasmid pAAVRep2Cap2 or pAAVRep2Cap5. Triple plasmid transfection using pAdHelper, instead of pBocaHelper, was used as positive control to compare the production efficiency between these two helper systems (Figure 1A). At 72 hr post-transfection, the cells were lysed with hypotonic buffer with surfactant sodium deoxycholate to release the rAAV particles. DNase-treated and clarified cell lysates were used for quantitation of the rAAV yields, which were indicated as DNase-resistant particle per cell (DRP/cell) by qPCR. The results showed that rAAV vectors were produced using pBocaHelper but with lower yields, which only accounted for 0.43% and 4.53% of the vector produced from pAdHelper for rAAV2/2 and rAAV2/5, respectively (Figures 1B and 1C). We then looked into AAV Rep and capsid protein expression in the rAAV-producing cells. Notably, AAV capsid protein expression was remarkably decreased in the pBocaHelper group, compared with the pAdHelper group (Figures 1D and 1E, VP panel, lane 1 versus 2). In addition to the barely detectable expression of capsid proteins, the large Rep78 and small Rep52 proteins were also decreased in the pBocaHelper-transfected cells (Figures 1D and 1E, Rep panel, lane 1 versus 2).

Taking all these results together, we concluded that the three HBoV1 gene products, NP1, NS2, and BocaSR, are capable of supporting rAAV production, as they do for WT AAV replication. However, the efficiency of supporting rAAV production is limited because pBocaHelper poorly induces expression of AAV Rep and capsid proteins from the helper pAAVRepCap during rAAV production.

BocaHelper Does Not Efficiently Transactivate the AAV P19 and P40 Promoters by AAV2 Rep78/68

AAV2 Rep78/68 transactivate viral P19 and P40 promoters in the presence of Ad co-infection or Ad helper gene expression provided by pAdHelper in HEK293 cells that express Ad *E1* gene.[31] The P19 and P40 promoters transcribe viral mRNAs to encode Rep52/40 and VP1/2/3, respectively. Since BocaHelper poorly facilitated Rep and capsid protein expression in rAAV production, we investigated AAV mRNA profiles by Northern blot analysis. The results showed that both the P19 and P40 promoters were poorly transactivated in the presence of pBocaHelper. There was >5 times less P19- and P40-transcribed mRNAs produced in the presence of pBocaHelper, as compared to pAdHelper (Figure 2, lane 2 versus 1 and 4 versus 3); this is reflected in the level of Rep52 and VP1/2/3 protein expression (Figure 1). These results suggest that the AAV2 P19 and P40 promoters are poorly transactivated by AAV2 Rep78/68 in HEK293 cells transfected with pBocaHelper and that this weakness in the production system leads to low expression of the AAV Rep and capsid proteins.

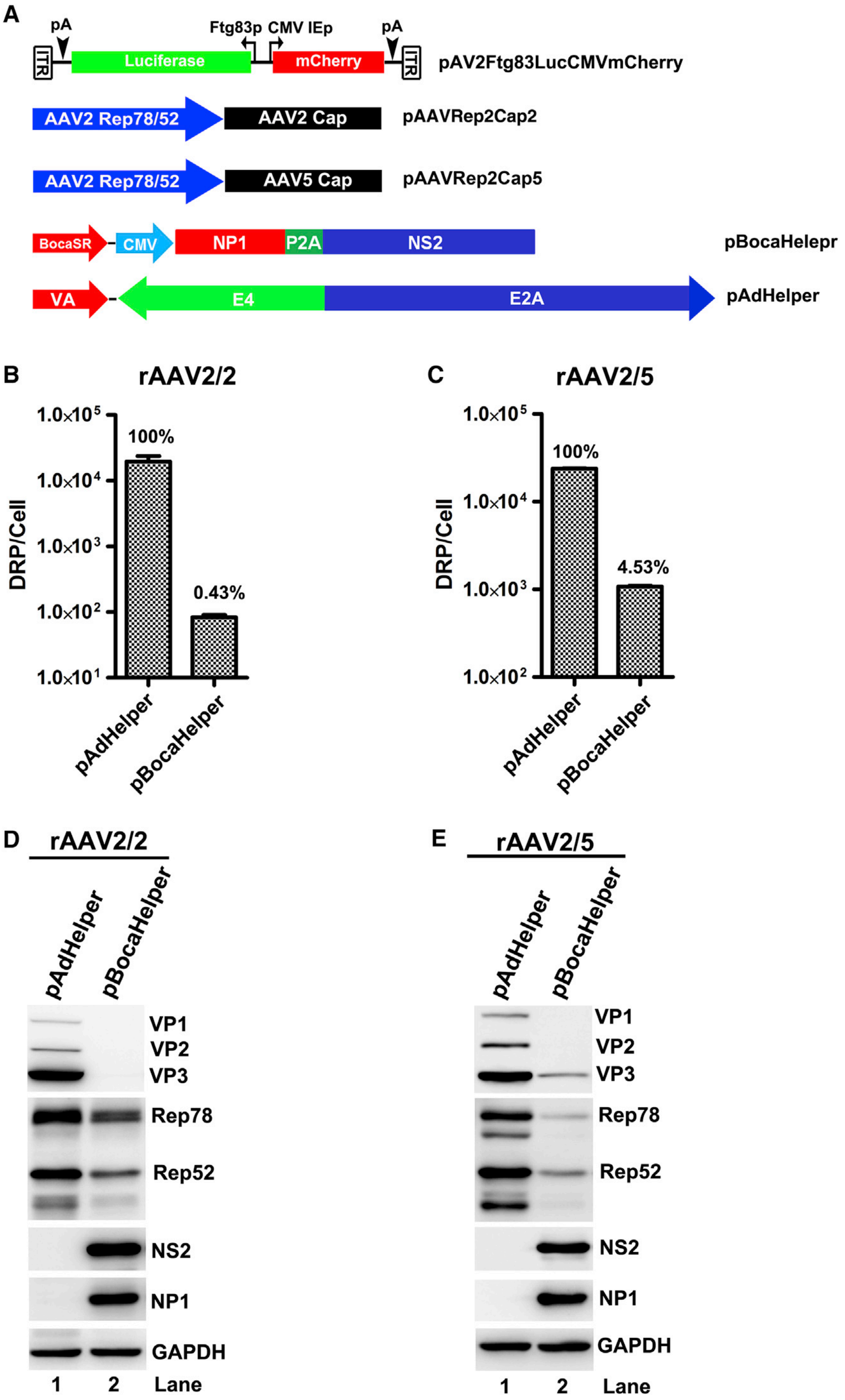

Figure 1. BocaHelper Facilitates the Production of rAAV2 and rAAV5 Vector

(A) Schematic diagram of plasmids. rAAV proviral genome, AAV helper (pAAVRep2Cap2 or pAAVRep2Cap5), adenovirus pAdHelper and human bocavirus pBocaHelper plasmids used in this study are diagramed. (B and C) Comparison of vector production helped with pAdHelper versus pBocaHelper. HEK293 cells were co-transfected with rAAV proviral vector (pAV2Ftg83lucCMVmCherry), with the AAV helper pAAVRep2Cap2 (B) or pAAVRep2Cap5 (C) plasmids and with pAdHelper or pBocaHelper. Cells were lysed at 72 hr post-transfection and DNase-resistant particles (DRP) were extracted and quantitated by real-time PCR. DRP of vector produced per cell is shown. (D and E) BocaHelper stimulates less AAV viral protein expression than pAdHelper does. The crude lysates from the experiments in (B) and (C) were mixed with 2× SDS loading buffer, respectively, boiled and resolved in 10% SDS-PAGE. Expression of AAV Rep78/52 and Cap (VP1/2/3) and HBoV1 (NS2 and NP1) as indicated were detected by western blotting. GAPDH was detected as a loading control.

BocaHelper Efficiently Facilitates rAAV Production when Sufficient Expression of Rep52 and Capsid Proteins Is Provided in *trans*

We next attempted to improve the expression of AAV capsid proteins. We used a strong constitutive promoter, the CMV IE gene promoter (CMV IE) for AAV2 *Cap* gene, and the AAV5 P41 promoter, of which the transcription activity is independent on AAV Rep proteins,[32] for AAV5 *Cap* gene (Figures 3A and 3D). AAV *Rep* genes were still expressed under its native P5 and P19 promoters. Western blot analysis showed that pCMVAV2Cap and p41AV5Cap expressed AAV capsid proteins without an obvious difference between in the transfections with pBocaHelper and pAdHelper (Figures 3B and 3E, VP panels, lane 1 versus 2). Accordingly, the pBocaHelper-mediated production of rAAV2/2 and rAAV2/5 vectors increased by 46.5-fold and 9.1-fold, respectively (i.e., 19.6% and 40.9% of the level when pAdHelper was used (Figures 3C and 3F).

AAV package is a highly regulated process, during which the Rep52 is responsible for packaging the single-stranded genome into assembled capsids via its DNA helicase activity.[33] We noticed that Rep52 was poorly expressed under its native P19 promoter in the pBocaHelper group (Figures 3B and 3E, Rep panels, lane 2 versus 1; Figures 1D and 1E, Rep panels, lane 1 versus 2), which leads us to speculate that Rep52 may be another limiting factor in BocaHelper-mediated rAAV production. To test this hypothesis, CMV-driven optimized Rep52 ORF (CMVoptRep52 cassette) was embedded in the pAV2Rep construct to increase Rep52 expression (Figures 3A and 3D). As shown in Figures 3B and 3E, the Rep52 expression was increased in the context of pAV2Rep-CMVoptRep52 with pBocaHelper. As expected, rAAV production

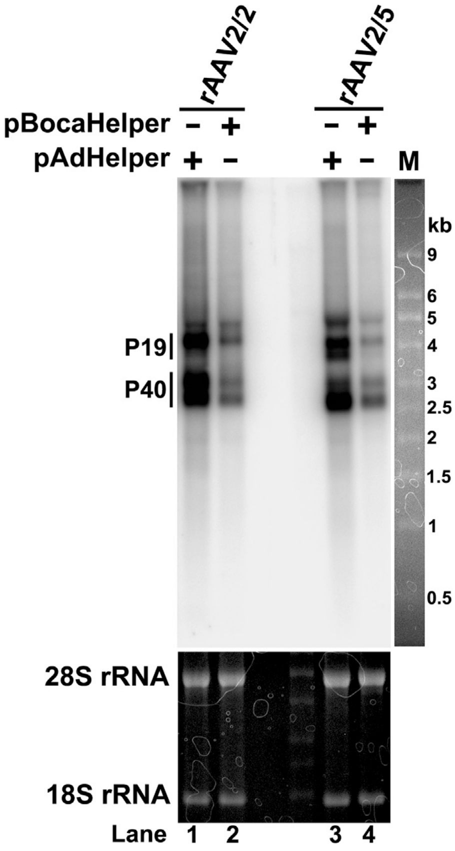

Figure 2. Northern Blot Analysis of AAV RNA Transcription

HEK293 cells were transfected as described in the experiments shown in (B) and (C) of Figure 1, and total RNAs were extracted and resolved in denature agarose gel. rAAV2/2 were probed with *Rep2Cap2* probe. rAAV2/5 were probe with *Rep2Cap5* probe. The 28S and 18S ribosome RNAs as loading controls were visualized by ethidium bromide staining. Bands of RNA transcripts by P19 and P40 promoters, respectively, are indicated.

efficiency was also further increased when the Rep52 expression was compensated (Figures 3C and 3F). rAAV2/2 and rAAV2/5 vectors produced with pBocaHelper reached to 53% and 77%, respectively, of that from pAdHelper. These results also indicated that there were other factors yet to be elucidated that limit rAAV production systems using pBocaHelper, in addition to Rep52 and capsid protein expression.

We purified the rAAV vectors produced using the HBoV1 or Ad helper system with the same procedure and found that the biological properties of rAAV produced with pBocaHelper remained the same as that produced with pAdHelper. We did not observe obvious differences in morphology between vectors produced with pBocaHelper versus pAdHelper under an electronic microscope (EM), and most of the virions are fully packaged with viral DNA (Figure S1A). The potencies of the two vector preparations were also compared side-by-side in HeLa and HEK293 cells. The two vectors transduced HeLa and HEK293 cells efficiently without significant difference (Figures S1B and S1C).

Poor Viral DNA Replication Is Another Limiting Factor for BocaHelper to Facilitate rAAV Production

We next sought to determine what other factor might limit rAAV production using a pBocaHelper-based system. To equally express all AAV Rep proteins, we introduced a CMV IE promoter to express AAV *Rep78* gene (Figures 4A and 4E). There were no differences in Rep78 and Rep52 expression between the pAdHelper and pBocaHelper groups (Figures 4B and 4F; Rep78 and Rep52). rAAV production was also compared among these two groups. The results showed that in the production of rAAV2/2 (Figure 4C) or rAAV2/5 (Figure 4G), there were still two-fold differences between pAdHelper and pBocaHelper groups. Because the optimized expression of all necessary AAV proteins in *trans* was not sufficient to achieve the same production titers using pAdHelper, we next evaluated the levels of rAAV2 DNA replication between the two groups. Southern blot analysis showed that pAdHelper facilitated rAAV2 DNA replication at a level of ~5 times higher than that from the pBocaHelper (Figures 4D and 4H).

Collectively, these results suggested that poor rAAV2 DNA replication was another limiting factor for the reduced vector production by pBocaHelper as compared to pAdHelper.

Ad *E2A* Gene Enhances BocaHelper-Facilitated rAAV Production by Increasing rAAV2 DNA Replication

In order to increase the above pBocaHelper-based rAAV vector packaging efficiency, we investigated whether these Ad genes in pAdHelper can further facilitate the packaging efficiency. Ad E2A protein is a single-stranded DNA (ssDNA)-binding protein localizes in AAV replication center that directly increases the processivity of AAV DNA replication;[34,35] Ad *E4* expresses E4Orf6 to accelerate AAV2 replication by the successful completion of second-strand synthesis.[36–38] It is also necessary to form a complex of E1b55K/E4Orf6 for the export of AAV mRNAs out of the nucleus.[36] E1b55K/E4Orf6 complex also promotes the degradation of Mre11/Rad50/Nbs1 complex (MRN) to liberate the obstacle of AAV replication.[39] VA RNA inhibits protein kinase R (PKR), which also increases AAV protein expression.[40] Adeno-associated RNA I (VAI) and BocaSR both are long noncoding RNA (lncRNA) and share similarity in the terms of nucleotide sequence and secondary structure.[41] We attempted to dissect these Ad helper functions in the context of the pBocaHelper-based system. By co-expression of each Ad component, we investigated its effect on rAAV2 DNA replication and vector production. pAV2Rep and pCMVAV2Cap, as illustrated in Figure 5A, were used to express AAV Rep and capsid proteins, respectively. Our results showed that pBocaHelper facilitated less rAAV2 DNA replication than that from pAdHelper, when *Rep* genes were provided by pAV2Rep (Figure 5B, lane 1 versus 2). Interestingly, rAAV2 DNA replication was obviously increased to a level similar to that from pAdHelper when Ad *E2A* gene was expressed (Figure 5B, lane 2 versus 3). The addition of Ad *E4* gene only slightly increased rAAV2 DNA replication (Figure 5B, lane 2 versus 4), but not the

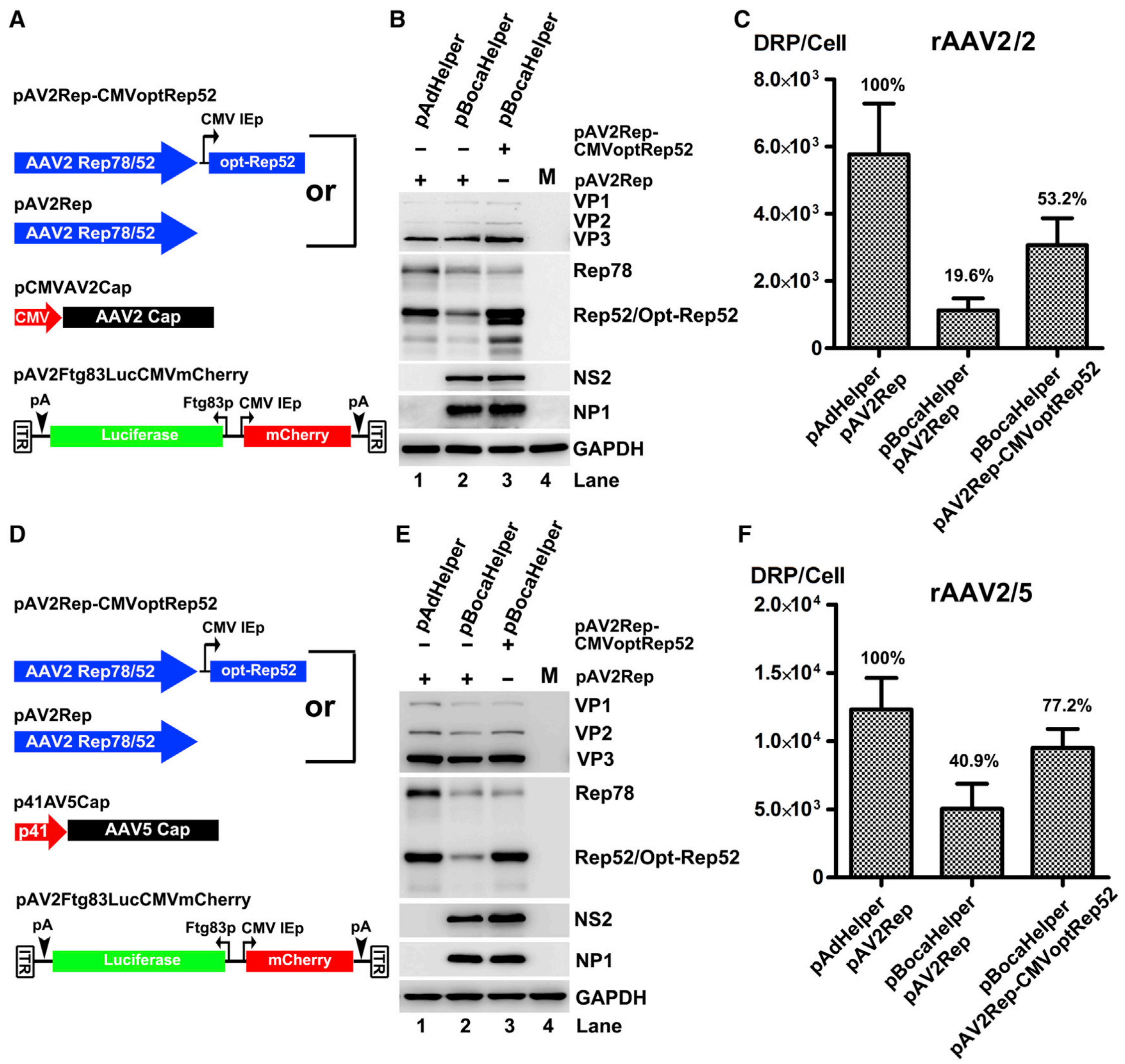

Figure 3. BocaHelper Supports a Comparative rAAV Package when AAV Rep52 and Capsid Proteins Are Compensated

(A and D) Plasmids used. pAV2Rep and pCMVAV2Cap (A) or p41AV5Cap (D) plasmids are schematically diagramed. The AAV helper functions were split into AAV2 Rep78/52 and AAV2 Cap (VP1/2/3) or AAV5 Cap with modification of its promoter or sequence to drive AAV2 Cap, AAV5 Cap, AAV2 Rep52 expression. (B and C) Viral protein expression and rAAV2/2 production. HEK293 cells were co-transfected with rAAV2 proviral vector, pAdHelper, or pBocaHelper and AAV2 helper plasmids (pCMVAV2Cap and pAV2Rep or pAV2Rep-CMVoptRep52). (B) Western blotting. At 72 hr post-transfection, expression of AAV2 and HBoV1 proteins during rAAV2/2 production. Viral proteins (AAV Rep and VP, and HBoV1 NP1 and NS2) were detected by western blotting. GAPDH was detected as a loading control. M, Mock transfected. (C) rAAV2 vector production. At 72 hr post-transfection, the cells were lysed for quantification of rAAV2/2 production. DRP per cell is shown. (E and F) Viral protein expression during rAAV2/5 production. HEK293 cells were transfected with rAAV2 proviral vector, pAdHelper or pBocaHelper, and p41AV5Cap and pAV2Rep or pAV2Rep-CMVoptRep52. (E) Western blotting. At 72 hr post-transfection, viral proteins were detected by western blotting. GAPDH was detected as a loading control. M, Mock transfected. (F) rAAV2/5 production. At 72 hr post-transfection, the cells were lysed for quantification of rAAV2/5 vector production. DRP per cell is shown.

addition of VA RNA expression (Figure 5B, lane 2 versus 5), suggesting the similar roles of these two lncRNAs in assisting rAAV replication. Importantly, the increment in rAAV2 DNA replication by the addition of *E2A* or *E4* gene was not due to the upregulation of AAV2 Rep expression (Figure 5C, lane 2 versus 3–5). In terms of the rAAV2/2 vector production, the results were exciting that the addition of each individual Ad helper gene all increased rAAV2/2 production significantly (over 3- to 4-fold) than BocaHelper alone, in particular, the addition of Ad *E2A* gene was the most effective. The combination of pBocaHelper and Ad *E2A* gene maximized rAAV2/2 production by nearly two times, compared with that from pAdHelper (Figure 5D). Similarly, we tested the combination of pBocaHelper and each one of the Ad helper genes for the production rAAV2/5 vector (Figure 5E). Ad *E2A*, *E4*, and *VA* genes all significantly increased rAAV2/5 production compared with those from pBocaHelper.

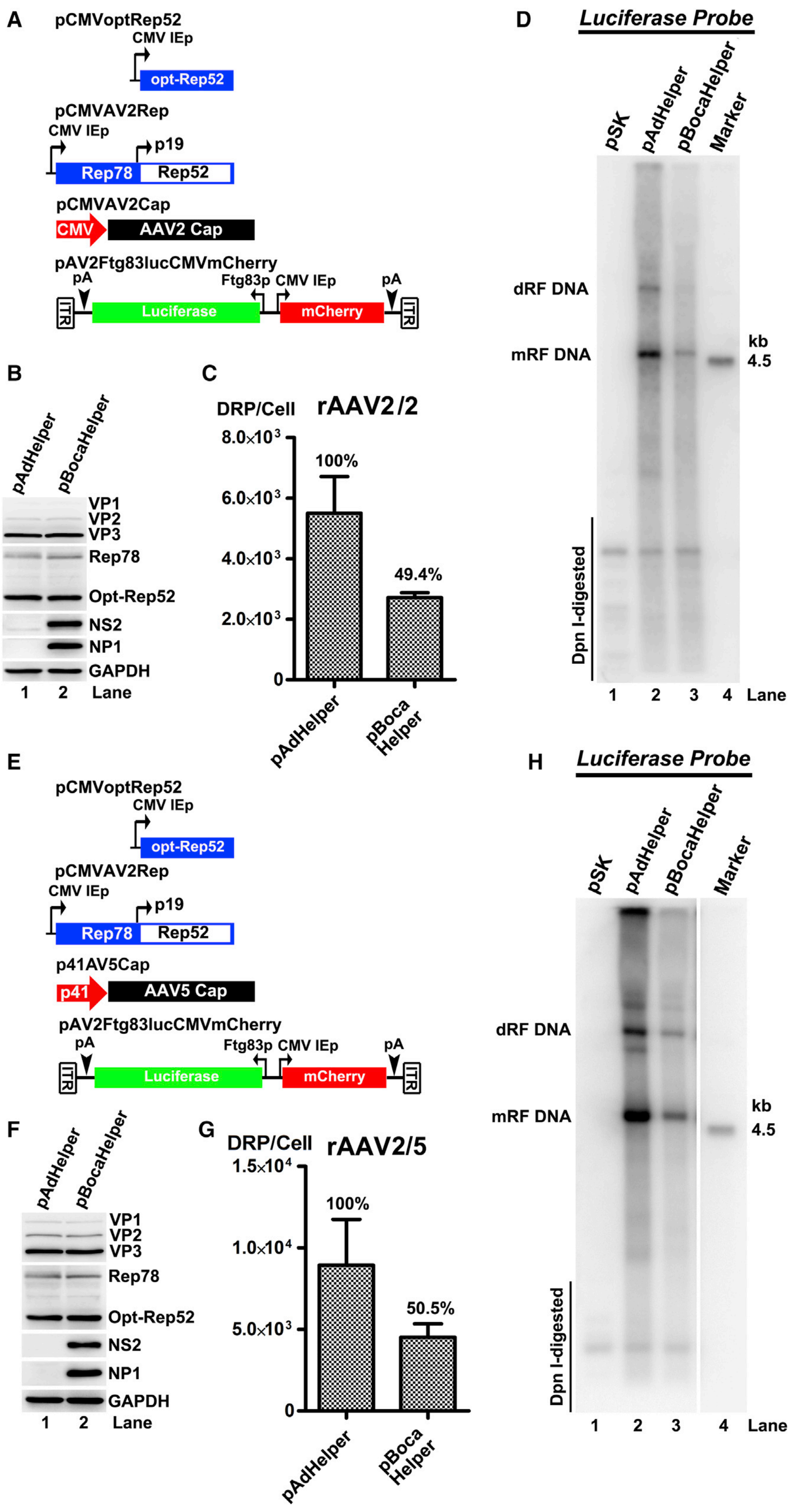

Figure 4. BocaHelper Facilitates rAAV Replication Less Efficiently Than pAdHelper in the Context of Constitutive Expression of AAV Helper Proteins

(A and E) Plasmids used. Plasmids pCMVAV2Rep, pCMV-OptRep52, pCMVAV2Cap (A), or p41AV5Cap (E) plasmids are schematically diagramed. (B–D) Comparison of viral protein expression, rAAV2/2 production, and rAAV DNA replication between pAdHelper and pBocaHelper. HEK293 cells were co-transfected with plasmids illustrated in (A). Cell lysates were collected at 72 hr post-transfection. (B) Western blot analysis of AAV Rep and capsid proteins. (C) Quantification of rAAV2/2 production. (D) Southern blot analysis of rAAV replication using a *luciferase* probe. (E and F) Comparison of viral protein expression, rAAV2/5 production, and rAAV DNA replication between pAdHelper and pBocaHelper. HEK293 cells were co-transfected with plasmids illustrated in (E). Cell lysates were collected at 72 hr post-transfection. (F) Western blot analysis of AAV2 Rep and AAV5 capsid proteins and HBoV1 NP1 and NS2 proteins. (G) Quantification of rAAV2/5 production. (H) Southern blot analysis of rAAV genome replication using a *luciferase* probe. dRF DNA and mRF DNA, dimer and monomer replicative form DNA, respectively.

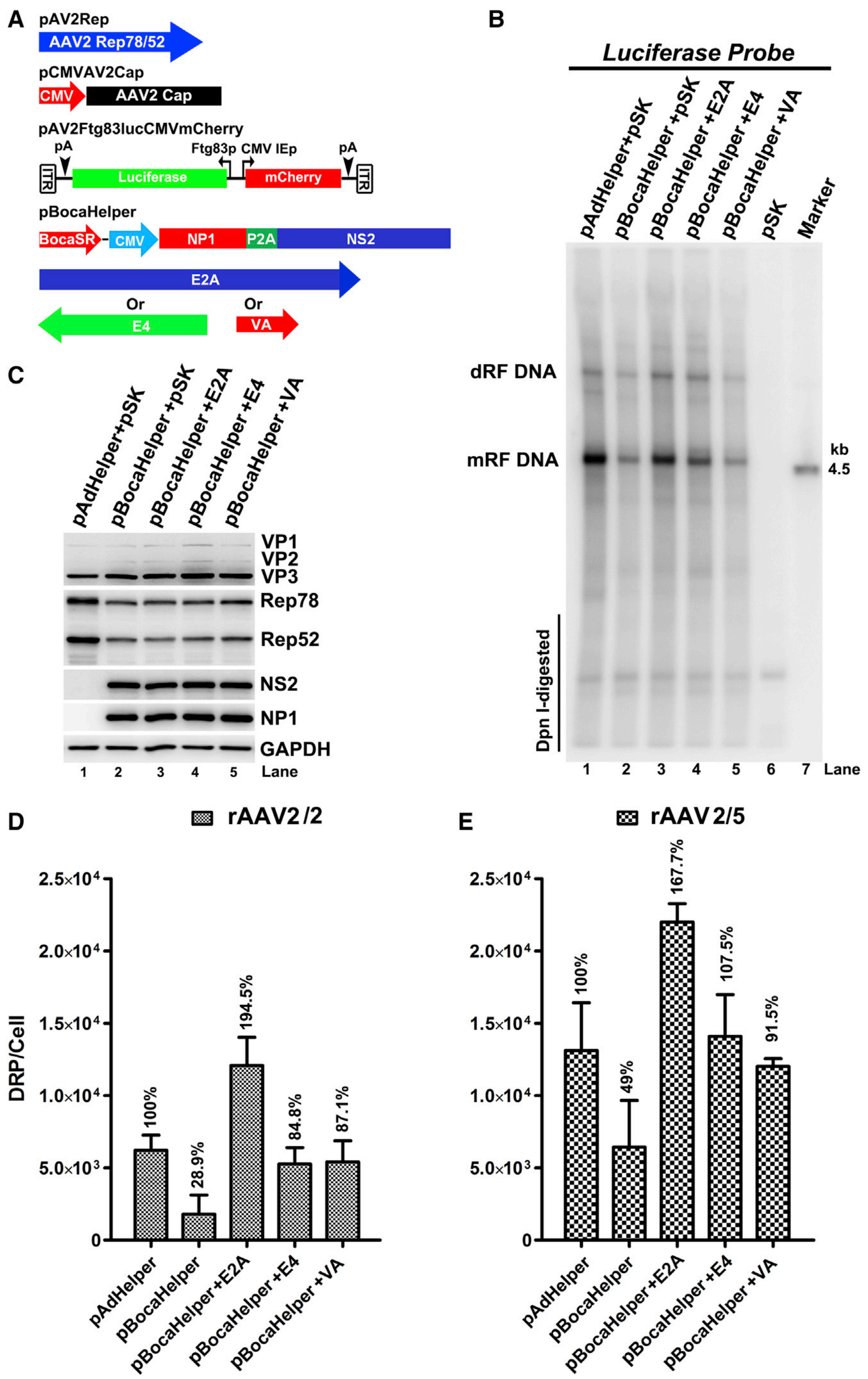

Figure 5. Adenovirus *E2A* Gene Expression Enhances BocaHelper in Facilitating rAAV Replication and Vector Production

(A) Plasmids used. Ad *E2A*, *E4*, and *VA* genes are transcribed from their authentic promoters. (B–D) HEK293 cells were co-transfected with plasmids as illustrated in (A). At 72 hr post-transfection, cells were collected for analyses. (B) Southern blot analysis of rAAV2 replication. Hirt DNA was extracted, and rAAV genome replication was analyzed by Southern blotting using a *luciferase* probe. (C) Western blot analysis of rAAV protein expression. AAV Rep and capsid proteins were probed with anti-Rep and anti-VP antibodies, respectively. HBoV1 NS2 and NP1 were also detected. GAPDH severed as a loading control. (D) rAAV2/2 production. Cells were lysed for rAAV2/2 vector quantification by qPCR. (E) rAAV2/5 vector production. HEK293 cells were co-transfected with plasmids as illustrated in (A), but not the pCMVAV2Cap, instead the p41AV5Cap. At 72 hr post-transfection, cells were collected and lysed for rAAV2/5 vector production. Vector yields are shown as DRP per cell.

Taken together, the results obtained from both rAAV2/2 and rAAV2/5 productions suggested that rAAV vector production helped by pBocaHelper can be further enhanced by Ad helper genes. The combination of *E2A* with pBocaHelper confers rAAV production the best, reaching a level significantly higher than that from the traditional pAdHelper in the five-plasmid transfection system (Figure 5A).

Ad and Human Bocavirus Helper Genes Have Synergistic Action in rAAV2 Production

Considering the enhancement effects of Ad genes, in particular *E2A* on BocaHelper-facilitated rAAV production that exceeded pAdHelper when capsid proteins were compensated, we wondered whether the addition of BocaHelper genes into pAdHelper can boost rAAV production in a simple three-plasmid-based vector production system. To this end, we constructed a dual with synergistic helper plasmid pABHelper (Figure 6A) that includes HBoV1 helper genes *NS2* and *NP1* in pAdHelper. We did not include the *BocaSR* gene, as it counteracts the function of VA RNA to some extent (data not shown). HEK293 cells were triple transfected with pAV2Ftg83LucCMVmCherry, pAAVRep2Cap2, and the dual helper pABHelper or pAdHelper for rAAV2/2 vector production. The results showed that pABHelper significantly increased the yield of rAAV2/2 by 1.8 times (Figure 6B). To further confirm the superior efficiency of pABHelper in rAAV production, we tested rAAV production in a large-scale preparation. The results confirmed that pABHelper enhanced rAAV2/2 production ~2 fold more than that with pAdHelper (Figure 6C). The underlying mechanism was also studied. We found that pABHelper enhanced rAAV genome replication by at least 2-fold (Figure 6D; monomer replicative form [mRF] DNA) but did not increase AAV Rep and capsid protein expression (Figure 6E).

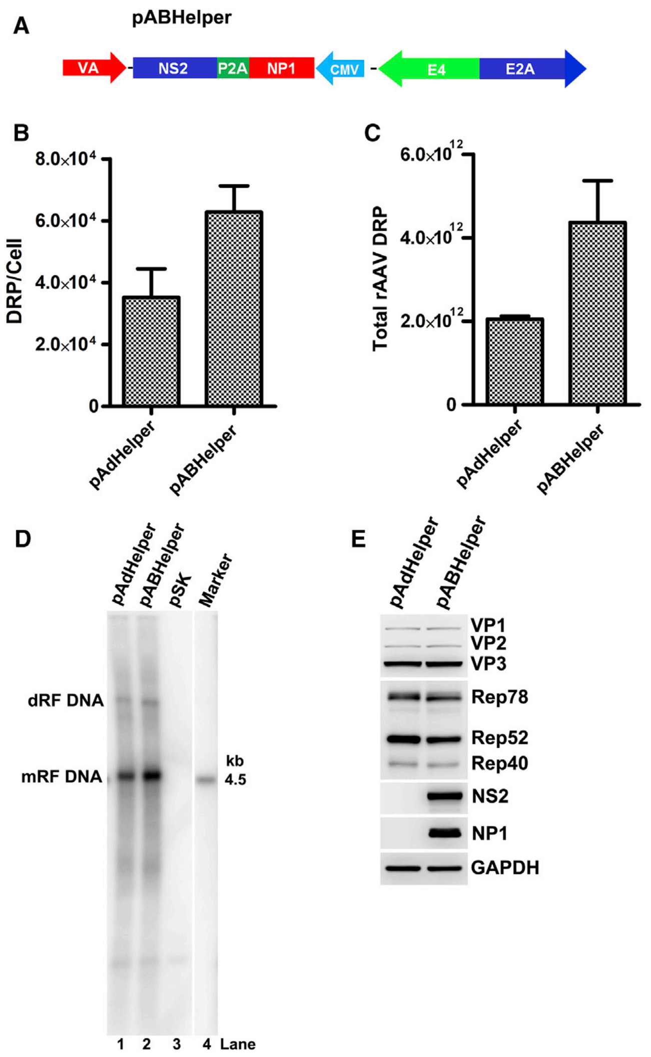

Figure 6. Human Bocavirus 1 *NP1* and *NS2* Genes Further Enhance rAAV Production That Is Facilitated by Adenovirus pAdHelper

(A) pABHelper. HBoV1 *NS2* and *NP1* genes, in addition to Ad *E2A*, *E4*, and *VA* genes, are diagramed with expression orientations shown as arrowheads. (B–D) HEK293 cells were co-transfected with pAV2Ftg83LucCMVmCherry, pAAVRep2Cap2, and pAdHelper or pABHelper. At 72 hr post-transfection, the cells were collected for further analyses. (B and C) rAAV2/2 production. (B) Vector production was determined by qPCR and is shown as DRP/cell. (C) Large vector preparations were made from 10 150-mm plates of HEK293 cells using pAdHelper versus pABHelper. Vector production was determined per preparation. (D) Viral DNA replication. Hirt DNA samples were extracted from transfected cells for Southern blotting using a *luciferase* gene probe. dRF DNA and mRF DNA, dimer, and monomer replicative form DNA, respectively. (E) Viral protein detection. Cell lysates were analyzed by western blotting for AAV Rep and capsid proteins and HBoV1 NS2 and NP1, as indicated. GAPDH was used as a loading control.

Taken together, these results indicated again that enhanced rAAV DNA replication is an approach to further increase rAAV vector yield.

DISCUSSION

In this study, we established a novel method to produce rAAV vector in HEK293 cells by expression HBoV1 helper genes *NS2*, *NP1*, and *BocaSR* from pBocaHelper. The yield of rAAV vector production from the pBocaHelper, in the context of satisfied expression of AAV helper genes *Cap* and *Rep52*, is approximately one time lower than that from the Ad helper plasmid pAdHelper. By extension of the study, we identified that the Ad *E2A* gene is able to additionally increase the yield of rAAV production with the helper of pBocaHelper. Finally, we constructed a dual helper plasmid pABHelper, which expresses HBoV1 NS2 and NP1 proteins, in addition to Ad E2A and E4 proteins and VA RNA. pABHelper is capable of enhancing rAAV production by ~2 times, compared with the traditional pAdHelper.

BocaHelper Does Not Facilitate Sufficient Transactivation of the P19 and P40 Promoters of the AAV Helper Construct and Replication of the rAAV Transgene

The AAV large Rep proteins (Rep78/68) transactivate AAV P19 and P40 promoters in the presence of Ad or expression of Ad helper genes.[27,42,43] Rep78/68, in the presence of Ad, can also enhance splicing of the P19- and P40-generated mRNAs, which are essential for the production of Rep52 and capsid proteins.[44] BocaHelper (*NS2*, *NP1*, and *BocaSR* genes) substitutes the function of the pAdHelper genes E2A, E4, and VA well in virus replication in the context of WT AAV[30] and the infectious clone of AAV2 (SSV9), but poorly in the production of rAAV vector (Figure 1). pBocaHelper produced 60% WT AAV2 of that by pAdHelper in HEK293 cells (data not shown). One of the two key factors is the low-level expression of the capsid and Rep52 proteins, which is largely due to the lower expression of VP and Rep mRNA transcripts in the presence of the pBocaHelper, suggesting the P19 and P40 promoters are not fully transactivated by the Rep78/68. This low level of AAV mRNA transcripts in rAAV production could explain the low yield of rAAV vector production from the BocaHelper, compared with the AdHelper. In contrast to VA RNA, BocaSR does not inhibit PKR.[30] We speculate that the low-level expression of Rep and capsid proteins may be in part due to the lack of PKR inhibition in the presence of BocaHepler. BocaHelper facilitates WT AAV replication in HEK293 cells as efficiently as AdHelper does in HEK293 cells.[30] The difference between WT AAV and rAAV is that both ITRs are missing in the AAV helper plasmid. ITRs not only function as replication and packaging elements, they also contain enhancer-like motifs and Rep-binding elements (RBEs), which positively regulate the transactivation of P19 and P40 promoters by Rep78/68 in *cis*.[45] Although the overall helper function of BocaHelper is weaker than AdHelper, the weakness was compensated when ITRs were present during WT AAV replication and/or production.

The second limiting factor is the less-supportive DNA replication by pBocaHelper than pAdHelper. When Rep and capsid protein expression is fully compensated, rAAV2 genome replicates at a level of ~5 times poorer with pBocaHelper than with pAdHelper, which results in only half of rAAV yield from pBocaHelper compared to that from pAdHelper.

Improving Recombinant Virus Replication Is a Considerable Direction in rAAV Production

The virus replication and productive infection are the outcome of virus host interaction. As a parasite of the cell, virus takes the advantage of host cellular transcription, translation, and replication machinery as well as cellular metabolism for its amplification. On the other hand, host cells sense virus infection, limit virus replication, and spread through innate immune and stress response. AAV is a defective virus, and its replication not only relies on host factors but also needs the necessary functions provided by helper virus. In the context of fully supplemented AAV and Rep and capsid proteins, BocaHelper is still compromised in the yield of rAAV production. We found that BocaHelper is a weak helper in supporting replication of the rAAV genome (Figure 4). We identified that additional expression of the Ad *E2A* gene can fully compensate the poor replication of the rAAV genome in the context of the BocaHelper and increase the yield of rAAV vector production superior to that produced by pAdHelper (Figure 5). It is a widely accepted notion that capsid protein expression limits rAAV production. Strategies that optimize AAV helper (especially capsid protein expression) boost rAAV production.[46,47] In this study, we confirmed that optimizing the helper virus functions also improved rAAV production. E2A is an ssDNA-binding protein of 72 kDa. It localized with AAV2 replication centers and probably bound to the single-strand AAV2 genome[34] and directly aided processivity of AAV DNA replication in an *in vitro* DNA replication assay.[35] We substituted with another ssDNA-binding protein of the HSV1, UL29, which has been reported as one of the essential HSV1 genes to facilitate AAV replication,[48] instead of the *E2A* gene in the system. An increase in viral DNA replication and rAAV production facilitated by UL29 was not observed (data not shown), suggesting that E2A enhances BocaHelper function not through its ssDNA-binding activity. Although the functions of the individual Ad helper gene have been studied extensively, it is still not fully understood what the exact function of E2A is in facilitating AAV DNA replication. We propose it is necessary to further study the function of E2A and how E2A enhances AAV DNA replication in the presence of HBoV1 NS2 and NP1 proteins, which are associated with the replicating viral genome during HBoV1 replication.[49]

Taking advantage of the synergistic stimulation function of the HBoV1 *NS2* and *NP1* genes and AdHelper genes, we made one dual helper plasmid pABHelper for rAAV production. The pABHelper enhances rAAV genome replication ~2 times, and thereafter vector production by ~2 times, while Rep and capsid protein expression are not significantly changed. Our study suggests that enhanced rAAV replication can further increase rAAV production, while capsid protein expression remains unchanged, which can be a future direction to increase rAAV production. The exact function of E2A in a synergistic function in enhancing rAAV genome replication together with HBoV1 NS2 and NP1 awaits further investigation.

Summary and Perspective

Our study established an alternative system for the production of rAAV2 using HBoV1 helper genes and identified a dual helper pABHelper for high-yield production of rAAV in HEK293 cells. Considering application of rAAV as a gene-therapy drug to humans and that large does are demanded, our pABHelper will be of significance in large-scale good manufacturing practice (GMP) production of rAAV vector using HEK293 cells. In addition, given the fact that expression of HBoV1 helper genes (*NP1*, *NS2*, *BocaSR*) facilitate WT AAV replication in HeLa cells at a level similar to that in HEK293 cells,[30] which do not express the Ad *E1* gene, we expect that our BocaHelper supports rAAV2 production in HeLa cells independent of Ad genes. Apparently, further application of the novel BocaHelper in rAAV production awaits us to explore.

MATERIALS AND METHODS

Plasmid Constructs

rAAV2 transfer plasmid pAVF5tg83luc-CMVmChery(4.6) was constructed by cloning the mCherry expression cassette CMV-mCherry ORF-bGHpA into pAVF5tg83luc.[9]

rAAV RepCap helper plasmids pAAVRep2Cap2 and pAAVRep2Cap5 have been described previously.[50]

pcDNA-FLAG-Rep78, which expresses N-terminal FLAG-tagged Rep78, was constructed by inserting the Rep78 ORF into pcDNA3.0 vector (Invitrogen) using EcoR V and Xba I sites.

pcDNA-Opt-Rep52 was constructed by inserting codon-optimized Rep52 (Opt-Rep52) ORF into pcDNA3.0 using the EcoR V and Xba I sites.

pcDNA-Opt-Rep52-P5Rep78, which expresses Opt-Rep52 and native Rep78, was constructed by inserting the AAV2 P5-Rep78-poly(A) expression cassette, which was obtained by PCR amplification of the cassette from pAV2Rep,[9] into pcDNA-Opt-Rep52 using Bgl II and Mfe I sites.

pCMVAV2Cap was constructed by inserting the CMV immediate early (IE) promoter (CMV nt 206 to 888 from pcDNA3; Invitrogen) into the Hind III site at AAV nt 1,882 of the P40VP AAV2 plasmid.[44] The 5′ splice site of the AAV2 intron in pCMVAV2Cap was optimized to be 5′-AG/GTA AGT-3′.

p41AV5Cap, which expresses AAV5 capsid proteins under its native promoter, has been described previously.[51]

pcDNA-Myc-NP1-P2A-NS2-BocaSR, the HBoV1 helper gene 3-in-1 plasmid, was constructed by inserting the NP1-P2A-NS2 cassette and HBoV1 BocaSR noncoding RNA (nt 5,129–5,360 of the HBoV1 genome) into pcDNA3.1-Myc vector using EcoR I and Xho I sites and Mfe I and Nru I sites, respectively. Porcine teschovirus-1 2A peptide (P2A) was used to separate the NP1 and NS2 ORFs.

pAdHelper, the Ad helper genes plasmid (pHelper) that contains the Ad5 *E2A*, *E4*, and *VA* gene, was purchased from Agilent (Santa Clara, CA). Individual Ad5 *E2A*, *E4*, and *VA* gene plasmids with their native

promoter were kind gifts from Drs. Gregory E. Tullis and David Pintel.[52]

pABHelper (Ad-Bocavirus combined helper), which contains the Ad5 *E2*, *E4*, and *VA* genes and HBoV1 *NP1* and *NS2* genes, were constructed by inserting the CMV-NP1-P2A-NS2-bGHpA cassette into pAdHelper using Cla I and Nsi I sites.

Cell Culture

HEK293 (ATCC CRL-1573) cells were purchased from ATCC and cultured in DMEM medium (Hyclone SH30022.01; GE Healthcare Life Sciences, Logan, UT) supplemented with 10% fetal bovine serum (FBS) (F0926, Sigma) at 37°C and 5% CO_2 atmosphere.

Plasmid DNA Transfection

HEK293 cells seeded in 60-mm dishes were transfected using the LipoD 293 reagent (SignaGen Laboratories, Gaithersburg, MD) following the manufacturers' instructions. The total amounts of plasmid DNA were kept constant (4 μg per 60-mm dish) in each group by supplementing empty vector to balance transfection efficiency.

Viral DNA Quantification

Titers of rAAV2/2 and rAAV2/5 were determined by real-time PCR quantification of DRPs. In brief, cells were lysed with sodium deoxycholate lysis buffer (25 mM Tris [pH 8.0], 150 mM NaCl, 0.5% sodium deoxycholate, 2 mM $MgCl_2$, 1 mM $CaCl_2$) and treated with Benzonase at a final concentration of 100 U/mL for 2 hr at 37°C for the DRP. Viral DNA from DRP was extracted using DNeasy Blood & Tissue Kit (QIAGEN) following the manufacturer's instructions for probe-based real-time PCR. The primers and probe for rAAV quantification, which target the luciferase gene, were as follows: forward primer 5′-TTT TTG AAG CGA AGG TTG TGG-3′, reverse primer 5′-CAC ACA CAG TTC GCC TCT TTG-3′, and PrimeTime probe, 5′-/6-FAM/ATC TGG ATA/ZEN/CCG GGA AAA CG-/3IABkFQ/-3′, purchased from IDT (Coralville, IA), were used. The standard curve for absolute quantification was generated from the rAAV proviral plasmid containing *luciferase* gene.

rAAV Vector Production

For large-scale rAAV production, HEK293 cells seeded in 150-mm dishes were transfected with total of 22 μg high-purity plasmids (at a molar ratio of 1:1:1 for rAAV *cis* transfer, pAAVRepCap, and pHelper) per dish using PEI MAX (cat. #24765, Polysciences) transfection reagent at a ratio of 1:3 (plasmid/PEI MAX). At 12 hr post-transfection, the medium was changed to 2.5% FBS-DMEM and cultured for an additional 60 hr before harvesting. For rAAV purification and quantification, we followed a previously published protocol.[9]

Western Blotting

Total proteins were extracted with Laemmli sample buffer, boiled, and resolved in 10% SDS-PAGE gel, transferred onto polyvinylidene fluoride (PVDF) membrane, blocked with nonfat dry milk, and probed with indicated antibodies, respectively. Anti-GAPDH (#A01622, GenScript), anti-AAV2 Rep (clone 303.9, #03-65169, American Research Products), anti-AAV2 VP (clone A69, #03-61057, American Research Products), and anti-c-Myc (clone 9E10, #SC-40, Santa Cruz) were purchased; anti-HBoV1 NS and anti-HBoV1 NP1 were homemade and have been described previously.[53]

Low-Molecular-Weight (Hirt) DNA Extraction and Southern Blotting

Hirt DNA was extracted using the modified Hirt method. In brief, cells were directly lysed with Hirt lysis buffer (10 mM Tris [pH 7.5], 10 mM EDTA, 0.6% SDS). The lysate was passed through a 25G needle several times. Then the samples were adjusted to 1.5 M NaCl and kept on ice overnight, followed by centrifugation at 13,000 × g for 15 min to precipitate a large DNA-protein complex. The supernatants were saved and treated with protease K at a final concentration of 1 mg/mL for 1 hr. Hirt DNA was purified using a DNA extraction kit (QIAGEN) following the manufacturer's instructions.

Southern blotting was performed according to our previously published method.[41,54] In brief, the Hirt DNA samples were digested with Dpn I, resolved into 1% agarose gel, blotted onto nitrocellulose membrane, and probed with ^{32}P-dCTP-labeled probes. For the detection of rAAV replication, a luciferase gene fragment was used as a probe template, which was excised from pAVF5tg83luc-CMVmChery(4.6) using Hind III and Xba I sites. Hybridization signals were captured by a storage phosphor screen and visualized on a Typhoon FLA 9000 biomolecular imager (GE Healthcare).

Northern Blotting

Northern blotting has been described previously.[55] In brief, total RNA was extracted and resolved in formamide-denatured agarose gel. 18S and 28S rRNAs were severed as loading control. For the detection of rAAV2/2 transcripts, the blot was probed with AAV2 probe. The template for AAV2 probe was the Xba I/Nde I-digested 4.3 kb of SSV9 [(psub201(-)],[56] which contains AAV2 Rep- and Cap-encoding sequence. For the detection of rAAV2/5 transcripts, the blot was probed with rAAV2/5 probe, which is the Age I and Xba I fragment of pAAVRep2Cap5. Hybridization and image acquiring followed the methods described in Low-Molecular-Weight (Hirt) DNA Extraction and Southern Blotting.

Statistics

Statistical analysis was performed using GraphPad Prism Version 7.0. Statistical significance was determined by using one-way ANOVA analysis. Error bars show mean ± SD from at least three independent experiments.

SUPPLEMENTAL INFORMATION

Supplemental Information includes one figure and can be found with this article online at https://doi.org/10.1016/j.omtm.2018.09.005.

AUTHOR CONTRIBUTIONS

Z.W. and F.C. conducted the experiments; Z.W., J.Q., Z.Y., and J.F.E. designed the experiments and wrote the paper.

CONFLICTS OF INTEREST

The authors have no conflicts of interest.

ACKNOWLEDGMENTS

We thank members of the Qiu lab for valuable discussions and suggestions. This study was supported by PHS grants AI070723, AI105543, and AI112803 from NIAID and the NIH to J.Q. and award YAN15XX0 from the Cystic Fibrosis Foundation to Z.Y. The funders had no role in study design, data collection and interpretation, or the decision to submit the work for publication.

REFERENCES

1. Cotmore, S.F., Agbandje-McKenna, M., Chiorini, J.A., Mukha, D.V., Pintel, D.J., Qiu, J., Söderlund-Venermo, M., Tattersall, P., Tijssen, P., Gatherer, D., and Davison, A.J. (2014). The family Parvoviridae. Arch. Virol. *159*, 1239–1247.
2. Samulski, R.J., and Muzyczka, N. (2014). AAV-Mediated Gene Therapy for Research and Therapeutic Purposes. Annu. Rev. Virol. *1*, 427–451.
3. Duan, D., Sharma, P., Yang, J., Yue, Y., Dudus, L., Zhang, Y., Fisher, K.J., and Engelhardt, J.F. (1998). Circular intermediates of recombinant adeno-associated virus have defined structural characteristics responsible for long-term episomal persistence in muscle tissue. J. Virol. *72*, 8568–8577.
4. Srivastava, A. (2016). In vivo tissue-tropism of adeno-associated viral vectors. Curr. Opin. Virol. *21*, 75–80.
5. Asokan, A., Schaffer, D.V., and Samulski, R.J. (2012). The AAV vector toolkit: poised at the clinical crossroads. Mol. Ther. *20*, 699–708.
6. Grimm, D., and Büning, H. (2017). Small But Increasingly Mighty: Latest Advances in AAV Vector Research, Design, and Evolution. Hum. Gene Ther. *28*, 1075–1086.
7. Grimm, D., and Zolotukhin, S. (2015). E Pluribus Unum: 50 Years of Research, Millions of Viruses, and One Goal–Tailored Acceleration of AAV Evolution. Mol. Ther. *23*, 1819–1831.
8. Tse, L.V., Klinc, K.A., Madigan, V.J., Castellanos Rivera, R.M., Wells, L.F., Havlik, L.P., Smith, J.K., Agbandje-McKenna, M., and Asokan, A. (2017). Structure-guided evolution of antigenically distinct adeno-associated virus variants for immune evasion. Proc. Natl. Acad. Sci. USA *114*, E4812–E4821.
9. Yan, Z., Keiser, N.W., Song, Y., Deng, X., Cheng, F., Qiu, J., and Engelhardt, J.F. (2013). A novel chimeric adenoassociated virus 2/human bocavirus 1 parvovirus vector efficiently transduces human airway epithelia. Mol. Ther. *21*, 2181–2194.
10. Cideciyan, A.V., Hauswirth, W.W., Aleman, T.S., Kaushal, S., Schwartz, S.B., Boye, S.L., Windsor, E.A., Conlon, T.J., Sumaroka, A., Roman, A.J., et al. (2009). Vision 1 year after gene therapy for Leber's congenital amaurosis. N. Engl. J. Med. *361*, 725–727.
11. Nathwani, A.C., Tuddenham, E.G., Rangarajan, S., Rosales, C., McIntosh, J., Linch, D.C., Chowdary, P., Riddell, A., Pie, A.J., Harrington, C., et al. (2011). Adenovirus-associated virus vector-mediated gene transfer in hemophilia B. N. Engl. J. Med. *365*, 2357–2365.
12. Gaudet, D., Méthot, J., Déry, S., Brisson, D., Essiembre, C., Tremblay, G., Tremblay, K., de Wal, J., Twisk, J., van den Bulk, N., et al. (2013). Efficacy and long-term safety of alipogene tiparvovec (AAV1-LPLS447X) gene therapy for lipoprotein lipase deficiency: an open-label trial. Gene Ther. *20*, 361–369.
13. Flotte, T.R., Trapnell, B.C., Humphries, M., Carey, B., Calcedo, R., Rouhani, F., Campbell-Thompson, M., Yachnis, A.T., Sandhaus, R.A., McElvaney, N.G., et al. (2011). Phase 2 clinical trial of a recombinant adeno-associated viral vector expressing α1-antitrypsin: interim results. Hum. Gene Ther. *22*, 1239–1247.
14. Gaudet, D., Méthot, J., and Kastelein, J. (2012). Gene therapy for lipoprotein lipase deficiency. Curr. Opin. Lipidol. *23*, 310–320.
15. Spencer, H.T., Riley, B.E., and Doering, C.B. (2016). State of the art: gene therapy of haemophilia. Haemophilia *22* (*Suppl 5*), 66–71.
16. Bennett, J., Wellman, J., Marshall, K.A., McCague, S., Ashtari, M., DiStefano-Pappas, J., Elci, O.U., Chung, D.C., Sun, J., Wright, J.F., et al. (2016). Safety and durability of effect of contralateral-eye administration of AAV2 gene therapy in patients with childhood-onset blindness caused by RPE65 mutations: a follow-on phase 1 trial. Lancet *388*, 661–672.
17. Feuer, W.J., Schiffman, J.C., Davis, J.L., Porciatti, V., Gonzalez, P., Koilkonda, R.D., Yuan, H., Lalwani, A., Lam, B.L., and Guy, J. (2016). Gene Therapy for Leber Hereditary Optic Neuropathy: Initial Results. Ophthalmology *123*, 558–570.
18. Dias, M.F., Joo, K., Kemp, J.A., Fialho, S.L., da Silva Cunha, A., Jr., Woo, S.J., and Kwon, Y.J. (2018). Molecular genetics and emerging therapies for retinitis pigmentosa: Basic research and clinical perspectives. Prog. Retin. Eye Res. *63*, 107–131.
19. Manno, C.S., Pierce, G.F., Arruda, V.R., Glader, B., Ragni, M., Rasko, J.J., Ozelo, M.C., Hoots, K., Blatt, P., Konkle, B., et al. (2006). Successful transduction of liver in hemophilia by AAV-Factor IX and limitations imposed by the host immune response. Nat. Med. *12*, 342–347.
20. Robert, M.A., Chahal, P.S., Audy, A., Kamen, A., Gilbert, R., and Gaillet, B. (2017). Manufacturing of recombinant adeno-associated viruses using mammalian expression platforms. Biotechnol. J. *12*, 10.
21. Kotin, R.M., and Snyder, R.O. (2017). Manufacturing Clinical Grade Recombinant Adeno-Associated Virus Using Invertebrate Cell Lines. Hum. Gene Ther. *28*, 350–360.
22. Clément, N., Knop, D.R., and Byrne, B.J. (2009). Large-scale adeno-associated viral vector production using a herpesvirus-based system enables manufacturing for clinical studies. Hum. Gene Ther. *20*, 796–806.
23. Wang, Q., Wu, Z., Zhang, J., Firrman, J., Wei, H., Zhuang, Z., Liu, L., Miao, L., Hu, Y., Li, D., et al. (2017). A Robust System for Production of Superabundant VP1 Recombinant AAV Vectors. Mol. Ther. Methods Clin. Dev. *7*, 146–156.
24. Grieger, J.C., Soltys, S.M., and Samulski, R.J. (2016). Production of Recombinant Adeno-associated Virus Vectors Using Suspension HEK293 Cells and Continuous Harvest of Vector From the Culture Media for GMP FIX and FLT1 Clinical Vector. Mol. Ther. *24*, 287–297.
25. Muzyczka, N. (1992). Use of adeno-associated virus as a general transduction vector for mammalian cells. Curr. Top. Microbiol. Immunol. *158*, 97–129.
26. Geoffroy, M.C., and Salvetti, A. (2005). Helper functions required for wild type and recombinant adeno-associated virus growth. Curr. Gene Ther. *5*, 265–271.
27. Xiao, X., Li, J., and Samulski, R.J. (1998). Production of high-titer recombinant adeno-associated virus vectors in the absence of helper adenovirus. J. Virol. *72*, 2224–2232.
28. Matsushita, T., Elliger, S., Elliger, C., Podsakoff, G., Villarreal, L., Kurtzman, G.J., Iwaki, Y., and Colosi, P. (1998). Adeno-associated virus vectors can be efficiently produced without helper virus. Gene Ther. *5*, 938–945.
29. Samulski, R.J., Sally, M., and Muzyczka, N. (1999). Adeno-associated virus vector. In The Development of Human Gene Therapy, T. Friedmann, ed. (Cold Spring Harbor Laboratory Press).
30. Wang, Z., Deng, X., Zou, W., Engelhardt, J.F., Yan, Z., and Qiu, J. (2017). Human Bocavirus 1 Is a Novel Helper for Adeno-Associated Virus Replication. J. Virol. *91*, e00710–17.
31. Graham, F.L., Smiley, J., Russell, W.C., and Nairn, R. (1977). Characteristics of a human cell line transformed by DNA from human adenovirus type 5. J. Gen. Virol. *36*, 59–74.
32. Qiu, J., Nayak, R., Tullis, G.E., and Pintel, D.J. (2002). Characterization of the transcription profile of adeno-associated virus type 5 reveals a number of unique features compared to previously characterized adeno-associated viruses. J. Virol. *76*, 12435–12447.
33. King, J.A., Dubielzig, R., Grimm, D., and Kleinschmidt, J.A. (2001). DNA helicase-mediated packaging of adeno-associated virus type 2 genomes into preformed capsids. EMBO J. *20*, 3282–3291.
34. Weitzman, M.D., Fisher, K.J., and Wilson, J.M. (1996). Recruitment of wild-type and recombinant adeno-associated virus into adenovirus replication centers. J. Virol. *70*, 1845–1854.

35. Ward, P., Dean, F.B., O'Donnell, M.E., and Berns, K.I. (1998). Role of the adenovirus DNA-binding protein in in vitro adeno-associated virus DNA replication. J. Virol. *72*, 420–427.

36. Samulski, R.J., and Shenk, T. (1988). Adenovirus E1B 55-Mr polypeptide facilitates timely cytoplasmic accumulation of adeno-associated virus mRNAs. J. Virol. *62*, 206–210.

37. Fisher, K.J., Gao, G.P., Weitzman, M.D., DeMatteo, R., Burda, J.F., and Wilson, J.M. (1996). Transduction with recombinant adeno-associated virus for gene therapy is limited by leading-strand synthesis. J. Virol. *70*, 520–532.

38. Ward, P. (2006). Replication of adeno-associated virus DNA. In The Parvoviruses, J. Kerr, S.F. Cotmore, M.E. Bloom, M.E. Linden, and C.R. Parrish, eds. (Hodder Arnold), pp. 189–211.

39. Schwartz, R.A., Carson, C.T., Schuberth, C., and Weitzman, M.D. (2009). Adeno-associated virus replication induces a DNA damage response coordinated by DNA-dependent protein kinase. J. Virol. *83*, 6269–6278.

40. Nayak, R., and Pintel, D.J. (2007). Adeno-associated viruses can induce phosphorylation of eIF2alpha via PKR activation, which can be overcome by helper adenovirus type 5 virus-associated RNA. J. Virol. *81*, 11908–11916.

41. Wang, Z., Shen, W., Cheng, F., Deng, X., Engelhardt, J.F., Yan, Z., and Qiu, J. (2017). Parvovirus Expresses a Small Noncoding RNA That Plays an Essential Role in Virus Replication. J. Virol. *91*, e02375–16.

42. Pereira, D.J., McCarty, D.M., and Muzyczka, N. (1997). The adeno-associated virus (AAV) Rep protein acts as both a repressor and an activator to regulate AAV transcription during a productive infection. J. Virol. *71*, 1079–1088.

43. Pereira, D.J., and Muzyczka, N. (1997). The adeno-associated virus type 2 p40 promoter requires a proximal Sp1 interaction and a p19 CArG-like element to facilitate Rep transactivation. J. Virol. *71*, 4300–4309.

44. Qiu, J., and Pintel, D.J. (2002). The adeno-associated virus type 2 Rep protein regulates RNA processing via interaction with the transcription template. Mol. Cell. Biol. *22*, 3639–3652.

45. McCarty, D.M., Christensen, M., and Muzyczka, N. (1991). Sequences required for coordinate induction of adeno-associated virus p19 and p40 promoters by Rep protein. J. Virol. *65*, 2936–2945.

46. Li, C., and Samulski, R.J. (2005). Serotype-specific replicating AAV helper constructs increase recombinant AAV type 2 vector production. Virology *335*, 10–21.

47. Cao, L., During, M., and Xiao, W. (2002). Replication competent helper functions for recombinant AAV vector generation. Gene Ther. *9*, 1199–1206.

48. Geoffroy, M.C., Epstein, A.L., Toublanc, E., Moullier, P., and Salvetti, A. (2004). Herpes simplex virus type 1 ICP0 protein mediates activation of adeno-associated virus type 2 rep gene expression from a latent integrated form. J. Virol. *78*, 10977–10986.

49. Shen, W., Deng, X., Zou, W., Engelhardt, J.F., Yan, Z., and Qiu, J. (2016). Analysis of cis and trans Requirements for DNA Replication at the Right-End Hairpin of the Human Bocavirus 1 Genome. J. Virol. *90*, 7761–7777.

50. Pillay, S., Zou, W., Cheng, F., Puschnik, A.S., Meyer, N.L., Ganaie, S.S., Deng, X., Wosen, J.E., Davulcu, O., Yan, Z., et al. (2017). AAV serotypes have distinctive interactions with domains of the cellular receptor AAVR. J. Virol. *91*, e00391–17.

51. Qiu, J., Nayak, R., and Pintel, D.J. (2004). Alternative polyadenylation of adeno-associated virus type 5 RNA within an internal intron is governed by both a downstream element within the intron 3′ splice acceptor and an element upstream of the P41 initiation site. J. Virol. *78*, 83–93.

52. Nayak, R., and Pintel, D.J. (2007). Positive and negative effects of adenovirus type 5 helper functions on adeno-associated virus type 5 (AAV5) protein accumulation govern AAV5 virus production. J. Virol. *81*, 2205–2212.

53. Chen, A.Y., Cheng, F., Lou, S., Luo, Y., Liu, Z., Delwart, E., Pintel, D., and Qiu, J. (2010). Characterization of the gene expression profile of human bocavirus. Virology *403*, 145–154.

54. Guan, W., Wong, S., Zhi, N., and Qiu, J. (2009). The genome of human parvovirus b19 can replicate in nonpermissive cells with the help of adenovirus genes and produces infectious virus. J. Virol. *83*, 9541–9553.

55. Qiu, J., Cheng, F., Burger, L.R., and Pintel, D. (2006). The transcription profile of Aleutian mink disease virus in CRFK cells is generated by alternative processing of pre-mRNAs produced from a single promoter. J. Virol. *80*, 654–662.

56. Samulski, R.J., Chang, L.S., and Shenk, T. (1987). A recombinant plasmid from which an infectious adeno-associated virus genome can be excised in vitro and its use to study viral replication. J. Virol. *61*, 3096–3101.

OMTM, Volume *11*

Supplemental Information

Development of a Novel Recombinant Adeno-Associated Virus Production System Using Human Bocavirus 1 Helper Genes

Zekun Wang, Fang Cheng, John F. Engelhardt, Ziying Yan, and Jianming Qiu

Supplemental Figure Legends

Fig. S1. Characterization of the biological properties of rAAV2/2 produced with the help of pBocaHelper.

(A) Electronic microscopy. Negative staining of recombinant AAV2 produced with the help of pAdHelper or pBocaHelper. **(B) The transduction efficiency of rAAV2 produced with pAdHelper or pBocaHelper.** HEK293 and HeLa cells were transduced at an MOI of 5,000 gc/cell. The expression of mCherry was viewed under a fluorescence microscope. **(C) Quantitative analysis the transduction efficiency by luciferase assay.** The transduced cells were lysed and the luciferase activities were measured. n.s., statistically no significant.

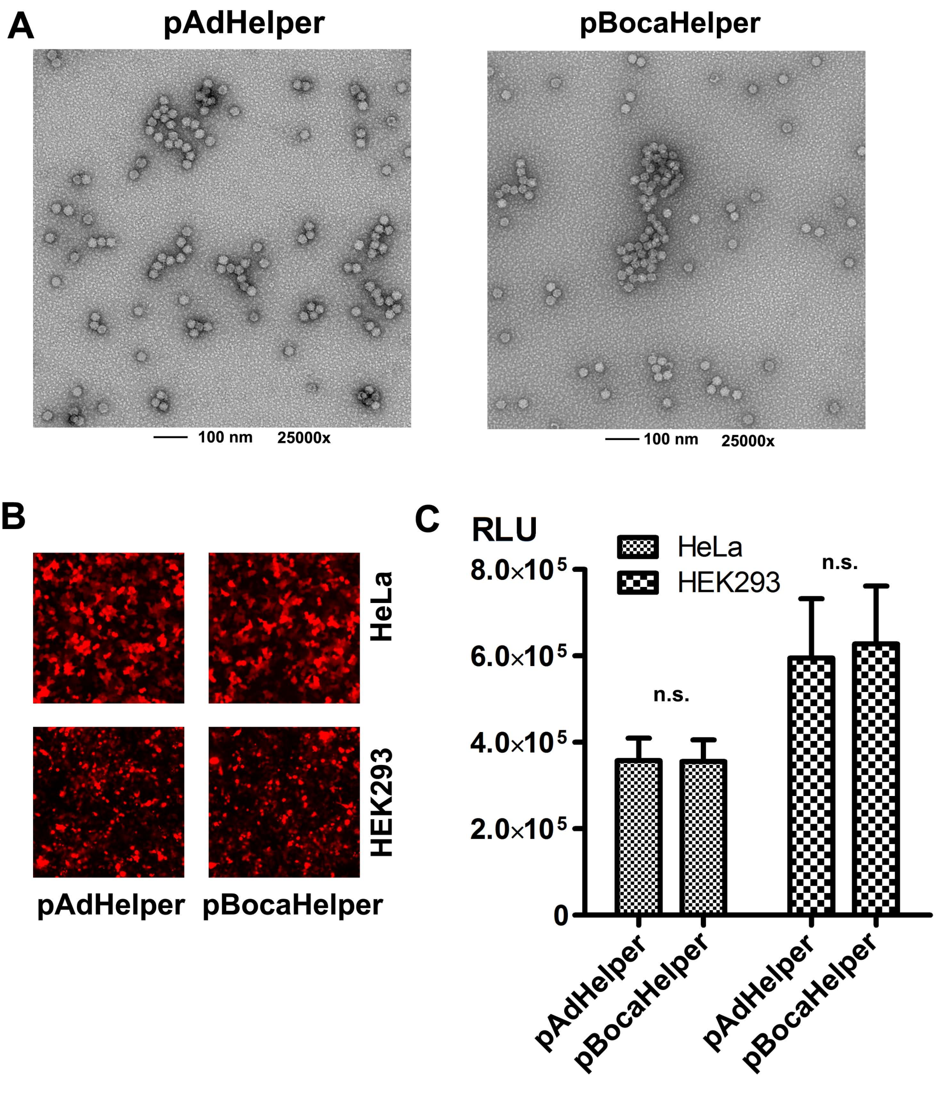

A
pAdHelper
pBocaHelper
100 nm 25000x
100 nm 25000x
B
HeLa
HEK293
pAdHelper
pBocaHelper
C
RLU
HeLa
HEK293
8.0×10⁵
6.0×10⁵
4.0×10⁵
2.0×10⁵
0
n.s.
n.s.
pAdHelper
pBocaHelper
pAdHelper
pBocaHelper

Figure S1
